# Prevention of non-communicable disease: best buys, wasted buys, and contestable buys

**DOI:** 10.1136/bmj.m141

**Published:** 2020-01-28

**Authors:** Wanrudee Isaranuwatchai, Yot Teerawattananon, Rachel A Archer, Alia Luz, Manushi Sharma, Waranya Rattanavipapong, Thunyarat Anothaisintawee, Rachel L Bacon, Tazeem Bhatia, Jesse Bump, Kalipso Chalkidou, Adam G Elshaug, David D Kim, Sumithra Krishnamurthy Reddiar, Ryota Nakamura, Peter J Neumann, Arisa Shichijo, Peter C Smith, Anthony J Culyer

**Affiliations:** 1 Health Intervention and Technology Assessment Programme, Bangkok, Thailand; 2 St Michael’s Hospital, Toronto, Canada; 3 University of Toronto, Toronto, Canada; 4 National University of Singapore, Singapore; 5 National Health Foundation, Bangkok, Thailand; 6 Department of Family Medicine, Ramathibodi Hospital, Mahidol University, Bangkok, Thailand; 7 Tufts Medical Center, Boston, USA; 8 Boston University, Boston, USA; 9 Public Health England, London, UK; 10 Harvard TH Chan School of Public Health, Boston, USA; 11 Centre for Global Development, London, UK; 12 Imperial College London, London, UK; 13 University of Sydney, Sydney, Australia; 14 Brookings Institution, Washington DC, USA; 15 Hitotsubashi University, Tokyo, Japan; 16 Tufts University School of Medicine, Boston, USA; 17 University of York, York, UK; 18 Imperial College Business School, London, UK

## Abstract

**Wanrudee Isaranuwatchai and colleagues** highlight the importance of local context in making decisions about implementing interventions for preventing non-communicable diseases

Increasing incidence of non-communicable diseases (NCDs) creates epidemiological and economic burdens everywhere and influences everyone regardless of sex and age.^1^ ^2^ Four main NCDs (cardiovascular diseases, diabetes, cancer, and chronic respiratory disease) account for over 12 million premature deaths worldwide annually.^3^ Mental ill health also imposes substantial economic burdens^1^ and should be included in policies for reducing the health and financial burdens of NCDs.

NCD policies have substantial implications for population health and national budgets. Countries therefore need to assess both the health and the financial aspects of these policies before implementation, especially in the context of the aim to achieve universal health coverage. We consider how to distinguish local best buys, wasted buys, and contestable buys among evidence based NCD interventions to improve setting of health priorities and offer suggestions for better decision making processes.

## Universal health coverage and prioritisation

Achieving universal health coverage requires clear thinking on the difficult choices about who is to be covered and for what interventions. The World Health Organization created a list of “best buys” to describe health interventions that are globally recommended for controlling NCDs.^3^ ^4^ They were selected using the following criteria: a demonstrated and quantified effect, cost effectiveness (≤$100 (£80; €90) per disability adjusted life year (DALY) averted in low and middle income countries), and implementability. A recent survey, however, found underuse of the best buy interventions, especially in low and middle income countries, and noted that insufficient action was being taken to reach the goal of reducing premature mortality from NCDs by a third by 2030 set out in the sustainable development goals.^5^ ^6^

One reason for this may be that much of the evidence on best buys does not come from low or middle income countries,^5^ and it is uncertain whether such global guidelines are helpful when setting priorities in these countries. Concerns exist about the transferability of study findings mostly from high income countries to other countries with different disease profiles, population characteristics, economic structures, health systems platforms, and other distinctive local characteristics. Moreover, no guideline exists on how to implement international research findings in these various settings, with varying implementation capacities.^4^

WHO and the Organisation for Economic Cooperation and Development have estimated that a fifth of total health spending in countries is wasted.^7^ ^8^ The waste exists for a multitude of reasons ranging from the lack of evidence needed to articulate better policy choices to governance related issues such as fraud and corruption. The waste is far more serious in low and middle income countries, where the overall disease burden is much higher but relatively small expenditures can have enormous impact if spent wisely. To improve priority setting at local level we distinguish best buys, wasted buys, and contestable buys recognising the importance of context.

## How context affects best buys

**Fig 1:**
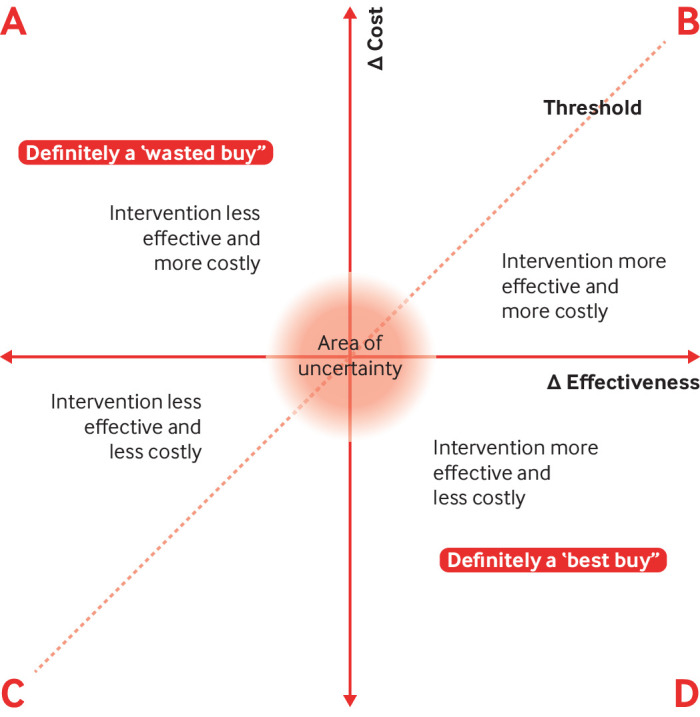
Cost effectiveness plane to show best buys and wasted buys. The broken line denotes the maximum amount the decision maker is willing to spend for additional health benefit. Interventions in quadrant A are clearly not cost effective compared with the current scenario and those in quadrant D are best buys. Decisions about interventions falling in quadrants B and C are less clear cut and will depend on the threshold set. The area of uncertainty indicates ranges of relatively small cost and benefit differentials where uncertainty may be particularly troublesome

The cost effectiveness plane is a visualisation of the differences in costs and outcomes of policy options, with costs plotted on the vertical y axis and effects (health gain) on the horizontal x axis (fig 1). An intervention that delivers no or little benefit or that might have adverse effects, and that uses up more resources than the current scenario is a wasted buy. An intervention is a contestable buy if it has no direct evidence of cost effectiveness in the country in which the intervention is being considered.

The principal criterion for both best buys and wasted buys is cost effectiveness. Although the methodological principles for cost effectiveness analysis are intended to be universal, their quantitative application often depends on local circumstances (context specificity).^9^ The threshold to separate the cost effective from the cost ineffective depends on how much a decision maker is willing to pay for additional health benefit and will therefore vary according to economic factors such as the budget for public expenditure.

A best buy in one setting could be a contestable or wasted buy in another setting. For example, although a fixed dose combination of antihypertensive and lipid lowering drugs was found to be the best buy for primary prevention of cardiovascular diseases among moderate risk population in high income countries,^10^ ^11^ Tanzania’s threshold ($610 per DALY at the time of study) was insufficient to warrant proceeding and the combination drug is a wasted buy in this middle income country.^12^ Evidence on diabetic screening in Indonesia and Thailand shows that screening is a best buy only for high risk groups aged ≥40 rather than from age 15, which had been the standard practice.^13^ ^14^ Screening people aged 15-39 age was a wasted buy, and the savings from focusing on the high risk groups released resources for other priorities.

Local context can also influence cost effectiveness.^15^ ^16^ For instance, tobacco taxation is widely considered to be a best buy and is included in WHO’s list.^4^ However, in India, the tax on cigarette products failed to substantially reduce tobacco use even though there was international evidence and strong political support for implementation. This was partly because 85% of smokers in India smoked bidi, a local tobacco that was not taxed.^17^ ^18^ Analysis of case studies of implementing NCD preventive policies in low and middle income countries^19^ ^20^ highlights important contextual economic and other considerations, including relevance to the community of interest; ethical acceptability; possibility of cross-sectoral collaboration; degree of community and stakeholder engagement; affordability, feasibility, and sustainability; and leadership, governance, compliance, and monitoring.^15^

## Beyond cost effectiveness

Clearly, what is best or wasted is more than just a question of effectiveness and cost. There are other ethical, cultural, political, and practical factors, some of which are modifiable. In some societies, religious taboos or conventions may have to be considered. In most, there will be concerns for greater health equality and reduced exposure to financial risk to supplement the efficiency criterion. The capacities of countries to use and implement research on cost effectiveness are also varied, which can easily result in mistaken judgments even about effectiveness and cost. Furthermore, interventions that affect NCDs often lie outside the health sector, including in education, transportation, or lifestyle. For example, though not in WHO’s best buy list, reducing physical inactivity through urban design is a pillar of NCD prevention. Bike sharing schemes have been advocated globally for many reasons related to health, economy, and the environment. In some countries, however, women are not fully able to use the schemes for cultural and religious reasons. Consequently, something initially lauded as a best buy becomes a contestable buy because of ambiguity about the intervention itself, the possible inequity it generates, and its gender bias. The resulting gender inequity in these contexts means that this intervention is not a best buy for the whole population and would need to be complemented with a policy to equally increase the physical activity of women.

NCD programmes need to balance national spending priorities fairly and efficiently against one another, safeguarding rights to health while having due regard for rights to education, security, decent housing, and so on. This decision requires high level priority setting at which budgets for health are determined. Even within the health sector, simple criteria may not suffice. For instance, seeking only to maximise health benefits can conflict with equity.^21^ Achieving equity tends to become costlier as policy reaches out to less accessible, marginalised groups. Exclusion of hard-to-reach populations raises important ethical questions regarding a just distribution of access to healthcare and of health itself.

Interventions that are not cost effective in one context might still be best buys in another. If this is suspected, they are “contestable” and may warrant more specific local study. Interventions that are in principle not cost effective might be best buys if they have other attractive attributes. For example, in settings with a commercial alcohol market rather than a tradition of home brewing, implementing a minimum unit price for alcohol could have more effect on health inequalities than simply raising alcohol taxes.^22^ A cost ineffective intervention may also be a best buy if it delivers sufficiently strong equity outcomes. Thailand implemented a policy of peritoneal dialysis first for patients with renal failure to ensure that people living in both urban and rural areas throughout the country could access the expensive, lifesaving treatment equally.^23^ ^24^

## Practical ways forward

NCD programme managers face challenges on several fronts, including information and political support. Keeping up with information in the health sector is not easy: 75 trials and 11 systematic reviews are published daily.^25^ Programme managers have to find ways of identifying relevant information. An expert hub, national or regional, could be used to gather, filter, and review relevant information as well as to support evidence assessment and appraisal processes.

**Fig 2:**
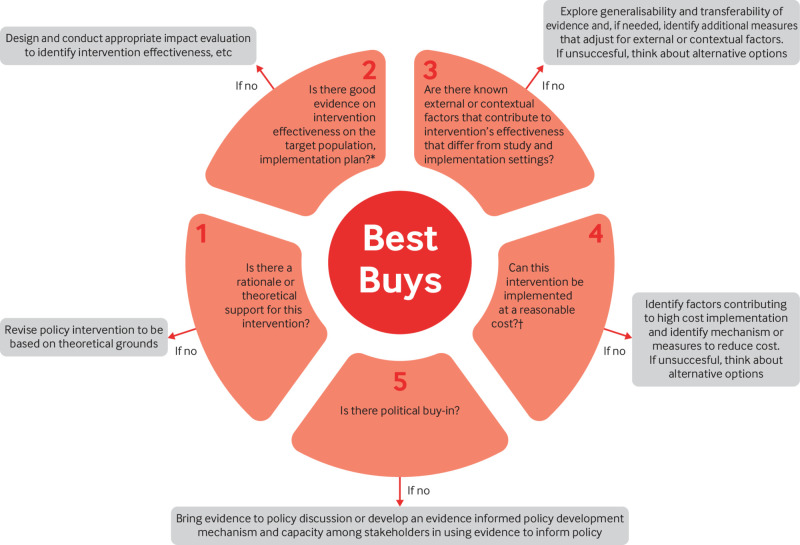
SEED tool for determining whether an intervention is likely to be worthwhile in a local context. The tool has two sections: the inner circle aims to assist NCD programme managers in thinking critically about the intervention and the outer boxes provide recommendations for strengthening the evidence base. *Implementation=dosage, frequency. duration, coverage, etc, †Compared with the cost of implementing a similar programme in other settings or the costs in the economic evaluation studies used to decide to implement the intervention

Health technology assessment (HTA) is not just a collection of technical methods, such as cost-effectiveness analysis, but also a way of thinking. It must include the academic disciplines of medicine, epidemiology, economics, management, and ethics. Systematic thinking for evidence based and efficient decision making (SEED) is one tool for determining whether an intervention is likely to be worthwhile in a local context (fig 2). It considers both the cost effectiveness agenda and the wider context (see web supplement). Since transferring economic evidence generated elsewhere to local settings is not straightforward,^14^ ^22^ ^23^ ^25^ we suggest using a scrutiny sequence starting with an initial environmental scan of the economic evidence (high or low income, organisation of healthcare, local prices, etc), followed by a data transferability assessment (see web supplement).^19^ ^26^ An institutional hub of national or regional technical expertise could support NCD managers in obtaining a clearer understanding of the local implications for health technology and transferability assessment.

Deciding whether any prospective intervention for NCDs is likely to be a best buy is tricky. The criteria may not be agreed; the evidence on which an appraisal can be based is rarely complete, accurate, or locally applicable; and the processes through which a decision about a possible best buy is made may be secretive, dominated by specific interest groups and incomprehensible to outsiders. Decision making needs to be credible to ensure policy acceptance and effective implementation. Any group likely to be affected by the decision needs to be able to examine the decision making process to discover whether the reasoning was sound, the value judgments were acceptable, and the evidence was appropriately identified and interpreted.^27^ The public will also want to be satisfied that those involved in the process were competent, that they sought to promote the public interest, and that those who were there to represent the public were appointed in a fair way and could be held to account.

Interventions tackling social determinants often require collaborations with other sectors, including government departments such as education, housing, and policing and the corporate sector. This may be a factor accounting for the underuse of best buys^28^ since nearly all systems of public administration are vertical and tend to make decisions in silos. Cross sectoral projects should be evaluated in the same way as all other uses of health sector funds.^29^ To be acceptable, the financial contribution of the health sector to a cross sectoral project should yield sufficient health benefits to make the project cost effective. Decisions ought to allow the full participation of sectoral partners. Systems characterised by short term planning and siloed budgets tend not to prioritise actions whose benefits are realised in the future and in sectors which did not initiate the original spend. Moreover, the structures of service delivery, whether, for example, an NCD unit is in or outside a ministry, could further inhibit the successful implementation of interventions. High level commitment and support at national and international levels is needed to scale up and accelerate the implementation of cross sectoral policies and interdepartmental collaboration for prevention and control of NCDs.^30^

Interventions to prevent NCDs are often complex, constantly changing, and unique to each jurisdiction. There is no single solution for all policy makers, but there are positive steps that can be taken to further their efforts. Whether an intervention for NCD prevention will be best, wasted, or contestable depends on the context. To understand the context is every bit as important as to understand the technologies of evaluation, and we have suggested some ways in which this might be done.

**Key messages**

- WHO’s list of best buy interventions aims to assist the global community’s fight on non-communicable diseases
- Best buys evaluated in one setting may not be cost effective in other settings
- Evaluation of context is essential to ensure resources are best deployed towards universal health coverage
- National or international hubs could help with this evaluation and build evidence to get cross sectoral support towards preventing non-communicable disease

## Web extra

Extra material supplied by authors

Web supplement: How to use the SEED tool and framework and checklist for transferability assessment

## Notes

Contributors and sources: All authors work in health technology assessment and health systems and policy research. All authors provided substantial contributions to the conception or design of the work and to the acquisition, analysis, or interpretation of data. They contributed to the draft and approved the final version. WI and YT are joint first authors. WI is the guarantor.

Provenance and peer review: Commissioned; externally peer reviewed.

This article is part of a series commissioned by *The BMJ*. Open access fees are paid for by the Bill and Melinda Gates Foundation, which had no involvement in the commissioning, peer review, or decision to publish.

## References

[1] Trautmann S Rehm J Wittchen HU The economic costs of mental disorders: Do our societies react appropriately to the burden of mental disorders? EMBO Rep 2016;17:1245-9. 10.15252/embr.201642951 27491723 PMC5007565

[2] World Health Organization Depression and other common mental disorders: global health estimates. WHO, 2017.

[3] World Health Organization. Noncommunicable diseases: Key facts. 2018 https://www.who.int/news-room/fact-sheets/detail/noncommunicable-diseases

[4] World Health Organization. Best buys and other recommended interventions for the prevention and control of noncommunicable diseases. 2017. https://apps.who.int/iris/bitstream/handle/10665/259232/WHO-NMH-NVI-17.9-eng.pdf;sequence=1

[5] Allen LN Pullar J Wickramasinghe KK Evaluation of research on interventions aligned to WHO ‘Best Buys’ for NCDs in low-income and lower-middle-income countries: a systematic review from 1990 to 2015. BMJ Glob Health 2018;3:e000535. 10.1136/bmjgh-2017-000535 29527342 PMC5841523

[6] World Health Organization Assessing national capacity for the prevention and control of noncommunicable diseases: report of the 2017 global survey. WHO, 2018.

[7] OECD Tackling wasteful spending on health. OECD, 2017.

[8] World Health Organization WHO global health expenditure atlas. WHO, 2014.

[9] Wilkinson T Sculpher MJ Claxton K The international decision support initiative reference case for economic evaluation: an aid to thought. Value Health 2016;19:921-8. 10.1016/j.jval.2016.04.015 27987641

[10] van Gils PF Over EA Hamberg-van Reenen HH The polypill in the primary prevention of cardiovascular disease: cost-effectiveness in the Dutch population. BMJ Open 2011;1:e000363. 10.1136/bmjopen-2011-000363 22189351 PMC3278482

[11] Liew D Park H-J Ko S-K Results of a Markov model analysis to assess the cost-effectiveness of a single tablet of fixed-dose amlodipine and atorvastatin for the primary prevention of cardiovascular disease in Korea. Clin Ther 2009;31:2189-203, discussion 2150-1. 10.1016/j.clinthera.2009.10.015 19922890

[12] Ngalesoni FN Ruhago GM Mori AT Robberstad B Norheim OF Cost-effectiveness of medical primary prevention strategies to reduce absolute risk of cardiovascular disease in Tanzania: a Markov modelling study. BMC Health Serv Res 2016;16:185. 10.1186/s12913-016-1409-3 27184802 PMC4869389

[13] Teerawattananon Y Kingkaew P Koopitakkajorn T Development of a health screening package under the universal health coverage: the role of health technology assessment. Health Econ 2016;25(Suppl 1):162-78. 10.1002/hec.3301 26774008 PMC5066643

[14] Rattanavipapong W Luz ACG Kumluang S One step back, two steps forward: an economic evaluation of the PEN program in Indonesia. Health Syst Reform 2016;2:84-98. 10.1080/23288604.2015.1124168 31514662

[15] Isaranuwatchai W Archer RA Teerawattananon Y Culyer AJ, eds. Non-communicable disease prevention: best buys, wasted buys and contestable buys. Open Book Publishers, 2019, 10.11647/OBP.0195. PMC7190374 31992592

[16] May CR Johnson M Finch T Implementation, context and complexity. Implement Sci 2016;11:141. 10.1186/s13012-016-0506-3 27756414 PMC5069794

[17] Nazar GP Chang KC Srivastava S Pearce N Karan A Millett C Impact of India’s National Tobacco Control Programme on bidi and cigarette consumption: a difference-in-differences analysis. Tob Control 2020;29:103-10. 10.1136/tobaccocontrol-2018-054621 30554161 PMC6952846

[18] Tobacco Institute of India. Tobacco taxation 2017. https://www.tiionline.org/industry-issues/taxation/.

[19] Health Intervention and Technology Assessment Program (HITAP). Call for case studies: examples of NCD prevention interventions in LMICs. 2018. http://www.globalhitap.net/wp-content/uploads/2018/09/Call-for-Case-Studies_PMAC-Commissioned-Work-.pdf.

[20] Ng E de Colombani P Framework for selecting best practices in public health: a systematic literature review. J Public Health Res 2015;4:577. 10.4081/jphr.2015.577 26753159 PMC4693338

[21] Rumbold B Baker R Ferraz O Universal health coverage, priority setting, and the human right to health. Lancet 2017;390:712-4. 10.1016/S0140-6736(17)30931-5 28456508 PMC6728156

[22] The University of Sheffield. Minimum unit pricing and strength-based taxation have larger impacts on health inequalities than increasing current alcohol taxes. 2016. https://www.sheffield.ac.uk/news/nr/alcohol-tax-taxation-minimum-pricing-1.552930.

[23] Tantivess S Werayingyong P Chuengsaman P Teerawattananon Y Universal coverage of renal dialysis in Thailand: promise, progress, and prospects. BMJ 2013;346:f462. 10.1136/bmj.f462 23369775

[24] Teerawattananon Y Dabak SV Khoe LC Bayani DBS Isaranuwatchai W To include or not include: renal dialysis policy in the era of universal health coverage. BMJ 2020;368:m82 10.1136/bmj.m82. 31992542 PMC7190365

[25] Bastian H Glasziou P Chalmers I Seventy-five trials and eleven systematic reviews a day: how will we ever keep up? PLoS Med 2010;7:e1000326. 10.1371/journal.pmed.1000326 20877712 PMC2943439

[26] Kim DD Bacon RL Neumann PJ Assessing the transferability of economic evaluations: a decision framework. In: Isaranuwatchai W Archer RA Teerawattananon Y Culyer AJ, eds. Non-communicable disease prevention: best buys, wasted buys and contestable buys. Open Book Publishers, 2019: 91-117, 10.11647/OBP.0195.06.

[27] Chalkidou K Culyer AJ Deliberative processes in decisions about best buys, wasted buys and contestable buys: uncertainty and credibility. In: Isaranuwatchai W Archer RA Teerawattananon Y Culyer AJ, eds. Non-communicable disease prevention: best buys, wasted buys and contestable buys. Open Book Publishers, 2019: 147-69, 10.11647/OBP.0195.09.

[28] Niessen LW Mohan D Akuoku JK Tackling socioeconomic inequalities and non-communicable diseases in low-income and middle-income countries under the Sustainable Development agenda. Lancet 2018;391:2036-46. 10.1016/S0140-6736(18)30482-3 29627160

[29] Jakab M Smith PC Cross-sectoral policies to address non-communicable diseases. In: Isaranuwatchai W Archer RA Teerawattananon Y Culyer AJ, eds. Non-communicable disease prevention: best buys, wasted buys and contestable buys. Open Book Publishers, 2019: 129-46, 10.11647/OBP.0195.08.

[30] World Health Organization Time to deliver: report of the WHO independent high-level commission on noncommunicable diseases. WHO, 2018.

