# Supplementary material for: Prevention of non-communicable disease: best buys, wasted buys, and contestable buys
Source: BMJ. 2020 Jan 28;368:m141. doi: 10.1136/bmj.m141 (PMC7190374; doi:10.1136/bmj.m141)
Supplement: Supplementary file 1 — Web supplement: How to use the SEED tool and framework and checklist for transferability assessment [file isaw54435.ww1.pdf]

Application of SEED tool to a Decision plus decision chart and checklist

Application of tool

The SEED tool (Figure 2 in main article) has two sections: the inner circle aims to assist NCD program managers in thinking critically about the intervention, while the outer boxes provide recommendations for strengthening the evidence base. In the inner circle, the tool highlights the importance of a sound theoretical basis, good quality evidence, transferability to the implementation setting, reasonable cost, and sufficient political commitment. The box below explores the application of the tool to a case study from Thailand: the implementation of diabetes mellitus and hypertension screening for people aged 15 years and older in 2002 for the country's universal coverage scheme. Though the policy was already implemented, the concepts from the tool were accounted for and applied in the course of the revision for the policy.

This box explores the application of the tool to a case study from Thailand: the implementation of a diabetes mellitus and hypertension screening for people aged 15 years and older in 2002 for the country's universal coverage scheme (UCS). Though the policy was already implemented, the concepts from the tool were accounted for and applied in the course of the revision for the policy.

Consideration 1: A sound theoretical basis	There were international guidelines that supported the implementation of the policy.
Consideration 2: Good quality evidence	With the theoretical basis, the policy was implemented. However, there was a lack of good quality evidence. The managers then sought to improve the evidence support (as recommended in the outer circle of the SEED Tool). A literature review was conducted to understand the most effective population-based interventions.
Consideration 2: Transferability to the implementation setting	After the reviews were conducted, expert consultation meetings were arranged to show the results and understand the priority issues that should be addressed by the policy. They found that ischaemic heart disease and diabetes mellitus were the top two considerations.
Consideration 4: Reasonable cost	The expert consultation results were used to inform local HTA studies on the cost-effectiveness and budget impact of the screening programs. They found that: diabetes mellitus screening was cost-effective only for the population aged 30 years and older and cardiovascular screening using the global risk score (e.g. blood pressure, cholesterol level, etc.) was value-for money for those aged 35 and older.
Consideration 5: Sufficient political commitment	The policy was implemented using Consideration 1. There was good political support for the policy. But, with political support for the use of evidence, the policy was revised to account for the recommendation of increasing the age of the population to from 15 years old to 30 and 35 years old for diabetes and cardiovascular screening, respectively, to ensure the effectiveness, value-for-money, and positive impact of the intervention.

Note: Modified from Teerawattananon Y, Kingkaew P, Koopitakkajorn T, Youngkong S, Tritasavit N, Srisuwan P, et al. Development of a Health Screening Package Under the Universal Health Coverage: The Role of Health Technology Assessment. Health economics. 2016 Feb;25 Suppl 1:162-78. PubMed PMID: 26774008. Pubmed Central PMCID: 5066643.

Decision Chart for Assessing Transferability and Evidence Review

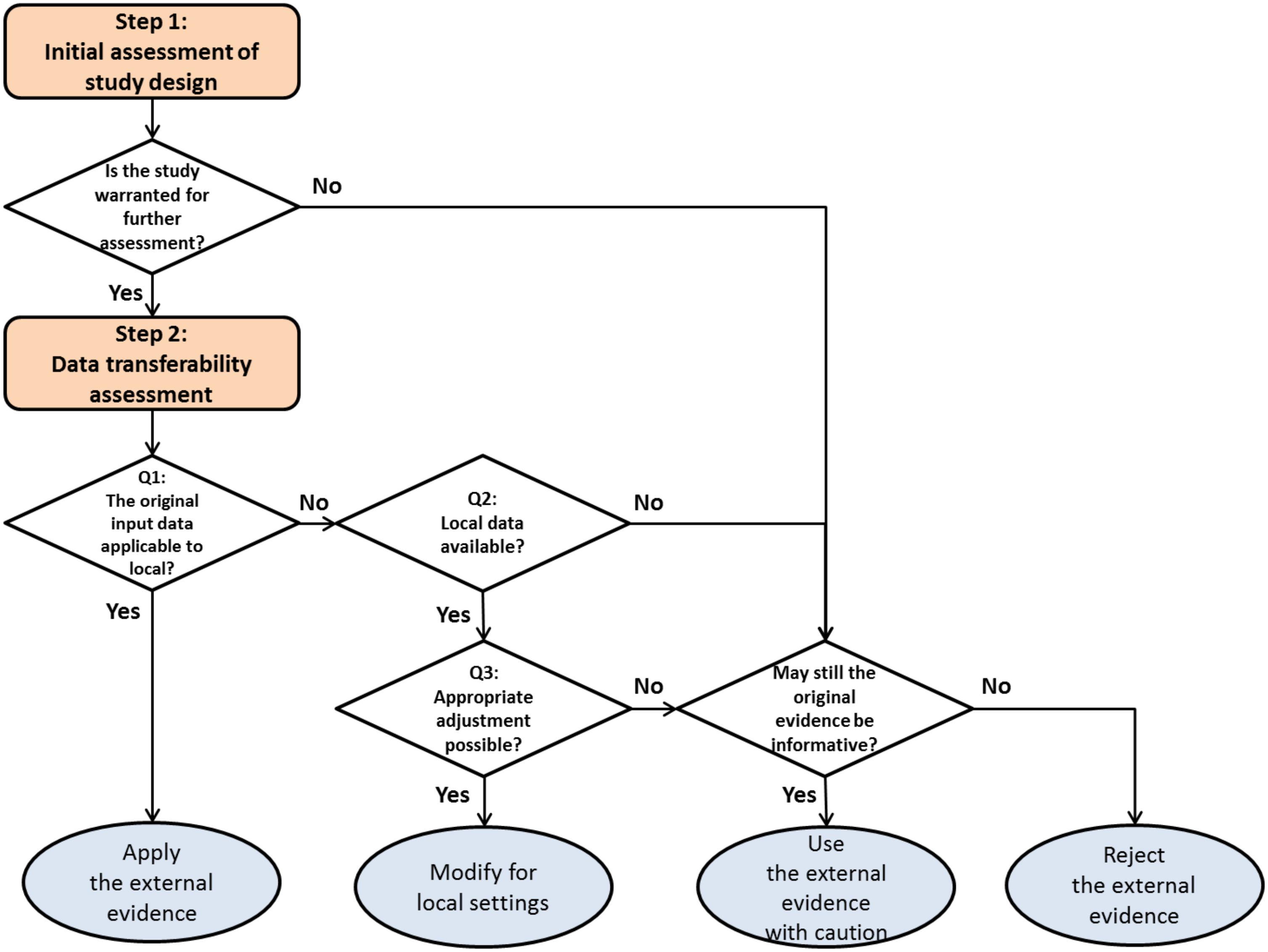

Transferability assessment checklist

<table>
<tr><th colspan="6">Step 1: Initial assessment of study design</th></tr>
<tr><td rowspan="2">Criteria</td><td colspan="4">Evaluation questions for each criterion</td><td rowspan="2">Decision Question:
Considering your evaluation for each criterion, is the original study warranted for the further assessment?</td></tr>
<tr><td colspan="2">Q1: Is the listed study characteristic aligned with local decision-making context? (If No, go to Q2)</td><td colspan="2">Q2: Is the original study still informative to the decision problem?</td></tr>
<tr><td>Study Perspective</td><td colspan="2"></td><td colspan="2"></td><td rowspan="5">A. No, reject the external evidence

B. No, but the external evidence can be used with caution

C. Yes, proceed to data transferability assessment</td></tr>
<tr><td>Intervention and its comparator(s)</td><td colspan="2"></td><td colspan="2"></td></tr>
<tr><td>Time horizon</td><td colspan="2"></td><td colspan="2"></td></tr>
<tr><td>Discounting</td><td colspan="2"></td><td colspan="2"></td></tr>
<tr><td>Study quality</td><td colspan="2"></td><td colspan="2"></td></tr>
<tr><th colspan="6">Step 2: Data transferability assessment</th></tr>
<tr><td rowspan="2">Major considerations</td><td colspan="4">Evaluation questions for each data input?</td><td rowspan="2">Decision Question:
Considering your evaluation for each criterion, is the original evidence transferable to your local setting?</td></tr>
<tr><td>Q1: Are the original input data applied to the local setting? (If No, go to Q2)</td><td>Q2: Is local data on the specific input available? (If Yes, go to Q3 If No, go to Q4)</td><td>Q3: Is appropriate adjustment for local data input possible? (If No, go to Q4)</td><td>Q4: Is the data input used in the original study still informative to the local context?</td></tr>
<tr><td>Baseline risk</td><td></td><td></td><td></td><td></td><td rowspan="5">A. No, reject the external evidence

B. No, but the external evidence can be used with caution

C. Yes, but only after appropriate adjustments for local data input

D. Yes, apply the external evidence as it is</td></tr>
<tr><td>Treatment effects</td><td></td><td></td><td></td><td></td></tr>
<tr><td>Unit costs/prices</td><td></td><td></td><td></td><td></td></tr>
<tr><td>Resource utilization</td><td></td><td></td><td></td><td></td></tr>
<tr><td>Health-state preference weight</td><td></td><td></td><td></td><td></td></tr>
</table>
